# Neurodevelopmental disorders in ICD-11 classification

**DOI:** 10.1192/j.eurpsy.2023.1547

**Published:** 2023-07-19

**Authors:** N. Zigic, I. Pajevic, M. Hasanovic, E. Avdibegovic, N. Aljukic, V. Hodzic

**Affiliations:** 1 Department of Psychiatry, University Clinical Center Tuzla, Bosnia and Herzegovina, University Clinical Center Tuzla; 2 Faculty of Medicine, Tuzla; 3 Pediatric ward, Health Center Banovići, Banovići, Bosnia and Herzegovina

## Abstract

**Introduction:**

The term “neurodevelopmental disorders” was first used in DSM-5. The ICD-11 retained this term, with some changes in the classification compared to DSM-5 and ICD-10.

**Objectives:**

To identify changes on neurodevelopmental disorders in three classifications.

**Methods:**

Review of neurodevelopmental disorders in ICD-10, DSM-5 and ICD-11.

**Results:**

Neurodevelopmental disorders applie to a group of disorders with early onset that affect cognitive and social development. ICD-10 doesn’t have a dedicated group for neurodevelopmental disorders and uses different terminology for specific conditions. DMS-5 replaced term “Mental Retardation” with less stigmatizing “Intellectual Disability”, while ICD-11 proposes term “Disorders of intellectual development”. They continue to be defined on basis of significant limitations in intellectual functioning and adaptive behaviour. In recognition of lack of access to locally appropriate standardized measures and due to importance of determining severity for treatment planning, ICD‐11 provide a comprehensive set of behavioural indicator tables. Another big change is made with hyperkinetic disorder, that is classified among behavioural disorders in child and adolescent age in ICD-10. In DSM-5 and ICD-11 is among neurodevelopmental disorders, replaced with term “Attention deficit hyperactivity disorder”. Pervasive developmental disorders that is consisted of eight different subtypes in ICD-10, in DSM-5 and ICD-11 is replaced with “Autism spectrum disorders” category. Guidelines for autism spectrum disorder have been substantially updated to reflect the current literature. According to ICD-11, autism spectrum disorders and ADHD may coexist in an individual, which is useful since there is good evidence that children with this comorbidity can benefit from stimulant medications. Finally, tic disorders in ICD-11 are classified under the Diseases of the nervous system, while in DSM-5 they are placed within neurodevelopmental disorders.

**Conclusions:**

ICD-11 doesn’t deviate significantly from DSM-5 when it comes to neurodevelopmental disorders, which is in accordance with the goal of WHO and APA to harmonize two psychiatric classifications.

**Disclosure of Interest:**

None Declared

